# Metacognitive Therapy Self-Help for Anxiety-Depression: Single-Blind Randomized Feasibility Trial in Cardiovascular Disease

**DOI:** 10.1037/hea0001168

**Published:** 2022-05

**Authors:** Adrian Wells, David Reeves, Calvin Heal, Peter Fisher, Patrick Doherty, Linda Davies, Anthony Heagerty, Lora Capobianco

**Affiliations:** 1 Faculty of Biology, Medicine and Health, School of Psychological Sciences, The University of Manchester; 2 Research and Innovation, Greater Manchester Mental Health NHS Foundation Trust, Manchester, United Kingdom; 3 NIHR School for Primary Care Research, Manchester Academic Health Science Centre, The University of Manchester; 4 Institute of Psychology, Health and Society, University of Liverpool; 5 Liverpool Clinical Health, The Royal Liverpool and Broadgreen University Hospital NHS Trust, Liverpool, United Kingdom; 6 Department of Health Sciences, University of York; 7 Centre for Health Economics, Division of Population Health, Health Services Research and Primary Care, Faculty of Biology Medicine and Health, School of Health Sciences, The University of Manchester; 8 Core Technology Facility, The University of Manchester School of Medical Sciences; 9 Manchester University NHS Foundation Trust, Manchester Royal Infirmary, Manchester, United Kingdom

**Keywords:** anxiety, depression, cardiac, cardiac rehabilitation, metacognitive therapy

## Abstract

***Background:*** One in three cardiovascular disease (CVD) patients experience significant anxiety and depression. Current psychological interventions have limited efficacy in reducing such symptoms and are offered as a face-to-face intervention that may be a barrier to accessing treatment. We evaluated the feasibility and acceptability of delivering assisted home-based self-help metacognitive therapy (home-MCT) to cardiac rehabilitation (CR) patients experiencing anxiety and depression. ***Method:*** One hundred eight CR patients with elevated anxiety and/or depression were recruited to a single-blind randomized feasibility trial across two United Kingdom National Health Service Trusts and were randomized to usual CR or usual CR plus home-MCT. The feasibility and acceptability of adding home-MCT to CR was based on credibility or expectancy ratings, recruitment rate, drop-outs, number of CR and home-MCT modules completed, and ability of outcome measures to discriminate between patients. The study was used to refine the sample size estimate for a full-scale trial. The quality of telephone support calls delivered by CR staff trained in MCT was assessed. ***Results:*** Home-MCT was found to be feasible and acceptable for the current CR patients with anxiety and depression. Recruitment and retention of participants was high, and attendance at CR was similar for both groups. Completion of home-MCT was high, but the quality of telephone support calls delivered was lower than expected. ***Conclusions:*** Home-MCT was acceptable and feasible to deliver to CR patients experiencing anxiety and depression, and the feasibility of conducting a full-scale trial of the intervention was established. Home-MCT may provide additional treatment options for cardiac patients experiencing psychological distress.

Cardiovascular disease (CVD) is one of the leading causes of death globally, responsible for 43% of deaths in Europe (Timmis et al., 2020) and 30% worldwide (GBD 2013 Mortality and Causes of Death Collaborators, 2013). CVD is also a major contributor to reduced quality of life (GBD 2017 DALYs and HALE Collaborators, 2018) and associated with significant health care expenditure, in the United Kingdom costing the NHS £42 million annually (NHS England, 2014), presenting a significant burden to health services (Bhatnagar et al., 2015). Evidence suggests that providing CVD patients with cardiac rehabilitation (CR) improves recovery, prevents further cardiac illness (Anderson et al., 2017) and improves health-related quality of life (Bethell et al., 2009). On average CR services consist of 8 weeks (Dalal et al., 2015) of group exercise classes, educational seminars, and in some cases psychological support. Anxiety and depression are common in CVD patients, with 18% of patients reporting moderate depression and 28% reporting moderate anxiety (Rao et al., 2020), despite this as few as 18% of CR services provide specialist psychological support (National Audit of Cardiac Rehabilitation, 2016).

While research highlights the physical health benefits of CR such as risk reduction in cardiovascular mortality (Anderson et al., 2016; Dalal et al., 2015), there has been a limited focus on mental health. National CR data shows only a 6% change in the proportion of patients moving from clinical anxiety or depression to normal anxiety or depression after CR (National Audit of Cardiac Rehabilitation, 2019). Given the relationship between elevated anxiety or depression and subsequent psychological and physical ill-health, improvements in psychological interventions delivered within CR services are necessary.

The British Association for Cardiovascular Prevention and Rehabilitation (BACPR) recommends a menu-based approach to CR stating that a choice of home-based programs should be offered (British Association for Cardiovascular Prevention and Rehabilitation, 2017). Home-based CR programs are structured programs that include exercise booklets and videos, monitoring, follow-up visits, letters or telephone calls from staff (Jolly et al., 2006). However, this approach has not been applied to psychological treatments to date, which have been primarily delivered and evaluated in face-to-face formats (Whalley et al., 2014). Self-help psychological support may provide significant advantages as it does not require delivery by a trained mental health professional, allows individuals to complete treatment at their own pace, may be cost-effective and may provide greater access to psychological support, especially in CR patients who may not be able or willing to attend face-to-face treatment or may be returning to work. At present, self-help psychological therapies for patients with a physical illness are largely based on relaxation techniques and cognitive behavioral therapy (Lambert et al., 2017), which demonstrate limited efficacy in improving psychological outcomes in this population. Lambert, et al. (2017) conducted a systematic review and meta-analysis including randomized controlled trials (*n* = 39) or quasi-randomized trial designs (*n* = 1). The review assessed the efficacy of self-help psychosocial interventions for anxiety and depression in patients with a physical illness. Self-help intervention formats varied and included workbooks or manuals, audio-recorded relaxation programs and telephone led emotional support. All interventions used therapeutic approaches based on cognitive behavioral therapy or included CBT exercises based on cognitive restructuring. There was a small but significant overall effect on anxiety outcomes posttreatment (SMD = −.17; 95% confidence interval, CI [−.32, −.02]) favoring self-help interventions, with a larger though still modest effect on outcomes for depression (SMD = −.35; 95% CI [−.55, .16]) at posttreatment. Over longer-term follow up (≥6 months postintervention) the results were mixed as outcomes for anxiety were no longer significant, while depression maintained a significant effect (SMD = −.33; 95% CI [−.57, −.08]). Lang et al. (2018) evaluated a self-help intervention for improving anxiety and depression in 50 patients with heart failure in the REACH HF trial. In this study, the home-based intervention utilized a manual that included cognitive-behavioral therapy and mindfulness-based exercises to improve symptoms of anxiety and depression. At 6-month follow up the intervention showed a small improvement in Hospital Anxiety and Depression Scale (HADS) anxiety scores (between group MD = .2) and a larger improvement in HADS depression scores (between group MD = 1.5; Lang et al., 2018). In summary, current home-based psychological options have variable and small effects on depression symptoms and limited effects on anxiety. There is clear need to develop more effective options that have a greater impact on anxiety and depression.

Recently, metacognitive therapy (MCT; Wells, 2009) has been shown to be effective and out-perform CBT in treating anxiety and depression in mental health settings (Callesen et al., 2020; Nordahl et al., 2018, Normann & Morina, 2018). With therapist delivered group-MCT also evaluated in a large-scale single-blind randomized trial of patients undergoing CR in the PATHWAY study (Wells et al., 2021). Group MCT was found to significantly improve both anxiety and depression symptoms when added to CR when compared with CR alone. Gains were maintained at 12-month follow-up with group-MCT maintaining superiority over CR alone on the majority of outcomes. In view of these results, MCT may be a good choice for delivery as a self-help intervention in patients with CVD. Furthermore, MCT is based on a model of psychological disorder symptoms that is transdiagnostic, aimed at identifying and modifying generic mechanisms that cut across anxiety and depression; thus, potentially simplifying the intervention.

The metacognitive model of psychological disorder (Wells & Matthews, 1994; Wells, 2019) on which MCT is based identifies a cognitive-attentional “syndrome” of difficult to control, perseverative negative thinking and persistent attention to threat as a central mechanism in emotion disorder. The syndrome is characterized by worry, rumination, threat-focused attention, and other maladaptive coping strategies, that arise from biases in metacognition, which includes metacognitive beliefs (i.e., beliefs about thinking, e.g., “I cannot control my health worries”). MCT focuses on effective regulation of worry, rumination and attention with the goal of modifying metacognitions behind inflexibility in these processes. MCT may be particularly well suited for patients with physical illnesses as unlike other psychological therapies it does not challenge the content of negative thoughts that are often realistic in medical patients (McPhillips et al., 2018).

While evidence supports the effectiveness of MCT in face-to-face delivery, to date a self-administered version of MCT has not been evaluated. Moreover, offering self-help psychological interventions are in line with BACPR recommendations for a more menu-based approach to CR. Self-help psychological interventions can be more flexible and convenient for patients, depending on their circumstances. CR attendance rates are generally lower among those with mobility issues, the poor, women and the BAME community; thus, self-help could increase accessibility (Galdas et al., 2018; National Audit of Cardiac Rehabilitation, 2017; Ruano-Ravina et al., 2016).

The National Institute of Health Research funded the PATHWAY program with the aim to examine the use and efficacy of metacognitive therapy in CVD patients during CR. In this article we report the first phase of the PATHWAY home-MCT RCT, which is a superiority trial comprising of an initial feasibility trial (reported here) followed by a main trial if indicated (trial protocol: Wells et al., 2018). Our research questions were: (a) Is Home-MCT feasible to deliver within CR services? (as indexed by recruitment and retention); (b) Is Home-MCT an acceptable treatment for treating anxiety and depression in CR patients?; (c) Is it feasible to conduct a randomized controlled trial (RCT) of the intervention within CR services and if so what is the sample size required for a full-scale definitive trial?; (d) Do the proposed trial outcome measures sufficiently discriminate between patients?

## Method

### Design

The PATHWAY Home-MCT feasibility study is a multicenter randomized controlled trial with 4- and 12-month follow-up comparing self-help MCT plus usual CR (intervention) versus usual CR alone (control). The trial received full ethical approval from the North West - Greater Manchester West Research Ethics Committee (Reference 16/NW/0786; IRAS ID 186990) and registered with a clinical trials database (ClinicalTrials.gov, identifier number NCT03129282). Participants were recruited from two NHS CR services (Bolton NHS Foundation Trust and Aintree University Hospitals NHS Foundation Trust). To be eligible for the study patients had to: (a) meet Department of Health and/or British Association for Cardiovascular Prevention and Rehabilitation CR eligibility criteria, (b) score eight or above on either the HADS anxiety subscale, depression subscale or both subscales, (c) be aged 18 or older, and (d) have a competent level of English language skills (able to read, understand, and complete questionnaires in English).

Before entering CR, patients are routinely sent the National Audit of Cardiac Rehabilitation (NACR) assessment pack, patients completed the HADS before attending their CR assessment. During the CR assessment, patients HADS questionnaires were screened and eligible patients were then assessed for full study eligibility. Eligible and interested patients were then provided with further information about the study. Potential participants were contacted by research assistants (RAs) to discuss the study further, arrange a mutually convenient time to obtain written informed consent, and complete baseline assessments. RAs, the chief investigator and the trial statisticians were blind to treatment allocation. Data monitoring was managed by the Greater Manchester Mental Health NHS Foundation Trust and project oversight was by an independent trial steering committee. The trial is reported in line with the CONSORT statement for the reporting of pilot and feasibility randomized controlled trials (Eldridge et al., 2016).

### Patient and Public Involvement

Patient and public involvement was used extensively throughout the study including grant development, participant recruitment, manual development, participant retention, and coproduction of the dissemination strategy. The PPI group provided feedback on earlier versions of the Home-MCT manual and supportive phone calls before the study.

### Randomization and Sample Size

Within each site, participants were stratified by sex and screening HADS score (anxiety score> = 8; depression score> = 8; both> = 8), then allocated to trial arms in a 1:1 ratio using randomized blocks of Size 4 and 6. Previous work has indicated that the impact of MCT may be greater in anxiety than depression (Normann & Morina, 2018), and previous studies have indicated the unhelpful causal processes such as rumination, are more prevalent or elevated in woman than men (Johnson & Whisman, 2013), as such HADS subscale scores at screening and sex were selected as stratification variables. Randomization was conducted via e-mail by a statistician at the Center for Biostatistics at the University of Manchester. Patients were informed of their group allocation via telephone by the trial manager. The chief investigator, trial statisticians, and research assistants were masked to patient allocation throughout data collection and analysis.

A target recruitment sample size of 108 (54 per arm) was based on having sufficient numbers of participants to reliably evaluate acceptability and feasibility, allowing for a “worst-case” attrition rate of 25%, resulting in a final *n* = 80, by which overall recruitment and retention rates for a full-scale trial could be estimated with an error of plus/minus 8% at most. This sample was also more than adequate for estimating variability in outcome measures for which samples of 40 are generally sufficient (Lancaster et al., 2004).

### Treatment

#### Treatment as Usual (CR-Alone)

CR programs include a combination of group exercise classes and educational seminars. Exercise classes and educational seminars are delivered on a weekly basis over 8 to 10 weeks for approximately 45–60 min each. Group educational seminars cover a range of topics including exercise, lifestyle management (i.e., healthy eating, medication management), relaxation (i.e., breathing exercises and progressive muscle relaxation), and stress management. CR sessions on stress management included psychoeducation on stress responses, relaxation techniques, and meditation.

#### Self-Help Metacognitive Therapy (Home-MCT Plus Usual CR)

Home-MCT is a self-help paper manual that consists of six modules. Modules focus on developing a case formulation, developing new strategies to regulate worry and rumination, and challenging metacognitive beliefs that maintain maladaptive patterns of thinking. One of the key techniques is the Spatial Attention Control Exercise (SpACE) that is regularly practiced. SpACE consists of following a set of brief instructions to engage, disengage, and shift attention to different spatial locations (left, right, front, and behind) and to listen for any sounds that might occur at those locations. It is used in conjunction with postpractice guided discovery through written questions aimed to increase metaawareness of control. Another strategy used to modify unhelpful metacognitions about uncontrollability of thinking is the use of worry or rumination delay, instructing participants how to step-back from trigger thoughts and postpone negative perseverative responses. Participants received three telephone support calls lasting up to 30 min each. Call one was an introductory call that introduced patients to the manual, format, and scheduled calls two and three. Call two and three were delivered after the completion of modules two and four and focused on reviewing the key learning points and provided support and guidance on the modules and implementing MCT strategies. Support calls followed a structured script and staff were reminded that their roles were to provide support and guidance on completing the home-MCT manual. Home MCT was offered in addition to usual CR.

### Therapists

Home-MCT support calls were delivered by CR staff who had received basic training in delivering the calls. There were seven therapists (all female, *M*_age_ = 47.2, *SD* = 6.9), three from Bolton and four from Liverpool. Therapists were predominantly CR nurses, with only one site including a physiotherapist. All staff had over 20 years of experience working in health care, but none had previous experience of delivering psychological therapy. Therapists completed a 2-day workshop delivered by the developer of MCT (Adrian Wells). Training included didactic teaching, role play, discussion, and studying the treatment manual. In addition, therapists piloted home-MCT support calls with five volunteers each (total of 1.5 hr of practice), after which an additional 1-day workshop was delivered, which focused on enhancing support skills. In total, therapists received 30 hr of training. No additional supervision or training was provided.

### Measures

#### Acceptability and Feasibility Outcomes

Three measures were used to assess the acceptability and feasibility of home-MCT. A credibility questionnaire was completed after participants had read the introduction to the manual but before starting the intervention and an adherence and exit questionnaire were administered after having completed the manual.

#### Credibility Questionnaire

The credibility questionnaire included three items that assessed perceptions of the manual rather than postintervention satisfaction or effectiveness and was completed after reading the introduction section of the home-MCT manual but before starting the first module. The questionnaire was adapted from the Credibility/Expectancy Questionnaire developed by Devilly and Borkovec (2000). The questionnaire assessed: (a) how logical Home-MCT seemed to the participant, (b) how successful they thought home-MCT would be in reducing levels of emotional distress, and (c) how confident the participant felt in recommending the intervention to someone experiencing similar problems. Each item was rated on a scale from 0 to 100.

#### Adherence and User-Friendliness Questionnaire

This questionnaire included six items and assessed the number of modules completed by participants (ranging from zero to six), the accessibility and usability of the home-MCT Manual, and to what extent participants felt that the supportive telephone calls were required. Completion of four modules or more was considered to be a clinically effective exposure to home-MCT (Wells et al., 2018). All questions, except for the number of modules completed, were rated on a scale from 0 to 100.This questionnaire was completed upon finishing the home-MCT manual.

#### Exit Questionnaire

The Exit Questionnaire consisted of two questions to collect specific details about the intervention, namely (a) “Which modules from the home-MCT manual have you completed?” and (b) “How much time have you spent to complete each module?” This questionnaire was completed at the end of the intervention, with the time-frame for returning this questionnaire being up to 4-months postrandomization.

### Outcome Measures

A battery of outcome and process questionnaires were completed by all participants at each assessment (i.e., baseline, 4 months follow-up and 12 months follow-up).

#### The HADS (Zigmond & Snaith, 1983)

The HADS is a 14-item measure of emotional distress that assesses symptoms of anxiety (seven items) and depression (seven items). Items are scored on a 4-point Likert scale with scores ranging from 0–21 for each subscale. Scores of 0 to 7 are categorized as normal, 8 to 10 as mild, 11 to 14 as moderate, and 15 to 21 as severe. The HADS has demonstrated good internal consistency in cardiac patients (Cronbach’s α anxiety = .84, depression = .76, total = .87; Martin et al., 2003).

#### The Impact of Events Scale—Revised (IES-R; Weiss & Marmar, 1997)

The IES-R is a 22-item measure assessing posttraumatic stress disorder symptoms across three subscales: intrusion, avoidance and hyperarousal. Items are scored on a 5-point Likert scale from zero (*not at all*) to four (*extremely*) on how distressing each symptom was during the past 7 days, with respect to the participant’s cardiac event. Scores can range between 0 to 88, with scores of 24 to 32 indicative of clinical concern and scores of 33 and above indicative of diagnosis of posttraumatic stress disorder. The IES-R has high internal consistency on total scores (Cronbach’s α = .95) and all subscales (Cronbach’s α intrusion = .90, avoidance = .86, hyperarousal = .85; Beck et al., 2008).

#### The Metacognitions Questionnaire-30 (MCQ-30; *Wells & Cartwright-Hatton,* 2004*)*

The MCQ-30 is a 30-item measure of metacognitive beliefs, which are assessed across five subscales: (a) positive metacognitive beliefs about the helpfulness of worry, (b) negative metacognitive beliefs concerning the uncontrollability and dangerousness of worry, (c) cognitive confidence, (d) cognitive self-consciousness, and (e) need to control thoughts. Items are scored on a 4-point Likert scale (1 to 4; *do not agree* to *agree very much*), whereby higher scores indicate greater maladaptive metacognitive beliefs. The internal consistency of the MCQ-30 has been shown to be high in cardiac patients across all subscales (Cronbach’s α positive metacognitive beliefs = .88, negative beliefs = .83, cognitive confidence = .91, cognitive self-consciousness = .81, cognitive control = .73) and the total score (Cronbach’s α = .91; Faija et al., 2020).

#### The Cognitive Attentional Syndrome-1 Revised (CAS-1R; Wells, 2015)

The CAS-1R is a 10-item measure to assess the degree of cognitive attentional syndrome activation in the past week across three subscales: coping strategies (including worry/rumination, attention), positive metacognitive beliefs and negative metacognitive beliefs. The CAS-1R has been adapted from the existing CAS-1 (Wells, 2009) for use in the PATHWAY trial. Items are rated on an 11-point Likert scale from 0 (*none of the time/not at all true*) to 100 (*all of the time/completely certain this is true*). Higher scores are an indication of greater use of unhelpful metacognitive beliefs or coping strategies. The CAS-1R has been found to have acceptable internal consistency in cardiac patients across two subscales (Cronbach’s α coping strategies = .88, negative metacognitive beliefs = .65) with low internal consistency for positive beliefs (Cronbach’s α positive metacognitive beliefs = .58). The measure has demonstrated good construct validity and found to predict anxiety and depression in cardiac patients (Faija et al., 2019).

#### EQ-5D-5L

The EQ-5D-5L (Herdman et al., 2011; Janssen et al., 2013) is a standardized measure of health status across five dimensions: mobility, self-care, usual activities, pain/discomfort, and anxiety/depression. Each dimension is rated on five options between no problems to extreme problems, along with an overall health measure on a visual analogue scale ranging from 0 (*worst health imaginable*) to 100 (*best health imaginable*). The EQ-5D-5L score can be calculated as a utility value. The utility value provides an index score of the five dimensions/levels, weighted by a set of societal preferences for different aspects of health. The utility score was estimated from the EQ-5D responses using the cross-walk mapping algorithm recommended by NICE at the time of the analysis (van Hout et al., 2012). Evaluation of the EQ-5D-5L demonstrated strong convergent validity and good discriminative abilities of the measure with cardiac patients (Dyer et al., 2010).

### Statistical Analysis

With a view to the potential for the study to act as an internal pilot for a subsequent definitive trial (i.e., the data would be combined with subsequent data if the trial were extended), data analysis was restricted to be primarily descriptive, with no between-group analysis of outcome measures, so as to maintain blinding to treatment allocation if extended. We assessed our sample size estimate for a full-scale trial and examined the ability of the outcome measures to discriminate between patients in terms of descriptive statistics.

We assessed the acceptability of adding Home-MCT to usual CR including assessing rates of recruitment into the study (number agreeing to participate out of those approached, and number recruited per month), withdrawal or drop-out by the primary endpoint of 4 months and by 12-month follow up (attrition rates), numbers of MCT modules completed (including time spent on each module), and number of CR sessions attended.

The feasibility of conducting a full trial was assessed with respect to completion of follow-up questionnaires (proportions of missing values), ability of the outcome measures to discriminate between patients (range of scores; floor or ceiling effects), and reestimation of the sample size for a definitive trial based on the findings of this study. We examined therapist competency in delivering supportive phone calls because these individuals were nonmental health specialists without prior experience of delivering psychological treatments. A quality rating checklist was used for this purpose, whereby two independent raters who were Level 1 MCT Registered Therapists rated recordings of supportive telephone calls. Only calls two and three were rated as these involved supporting patients on the content of Home-MCT. Call two was rated on the quality of supporting two treatment components by that stage of treatment (SpACE and worry delay), whereas call three was rated on quality of four components (SpACE, worry delay, challenging uncontrollability beliefs, and behaviors prescription). Telephone support calls were rated against a quality checklist where raters were asked to indicate a score of 0% (no evidence of component), 25% (implemented poorly), 50% (implemented moderately), 75% (implemented reasonably well), or 100% (implemented well). A total quality score was derived for each call by summing the total rating of elements completed in session.

## Results

### Participants

**Figure 1 fig1:**
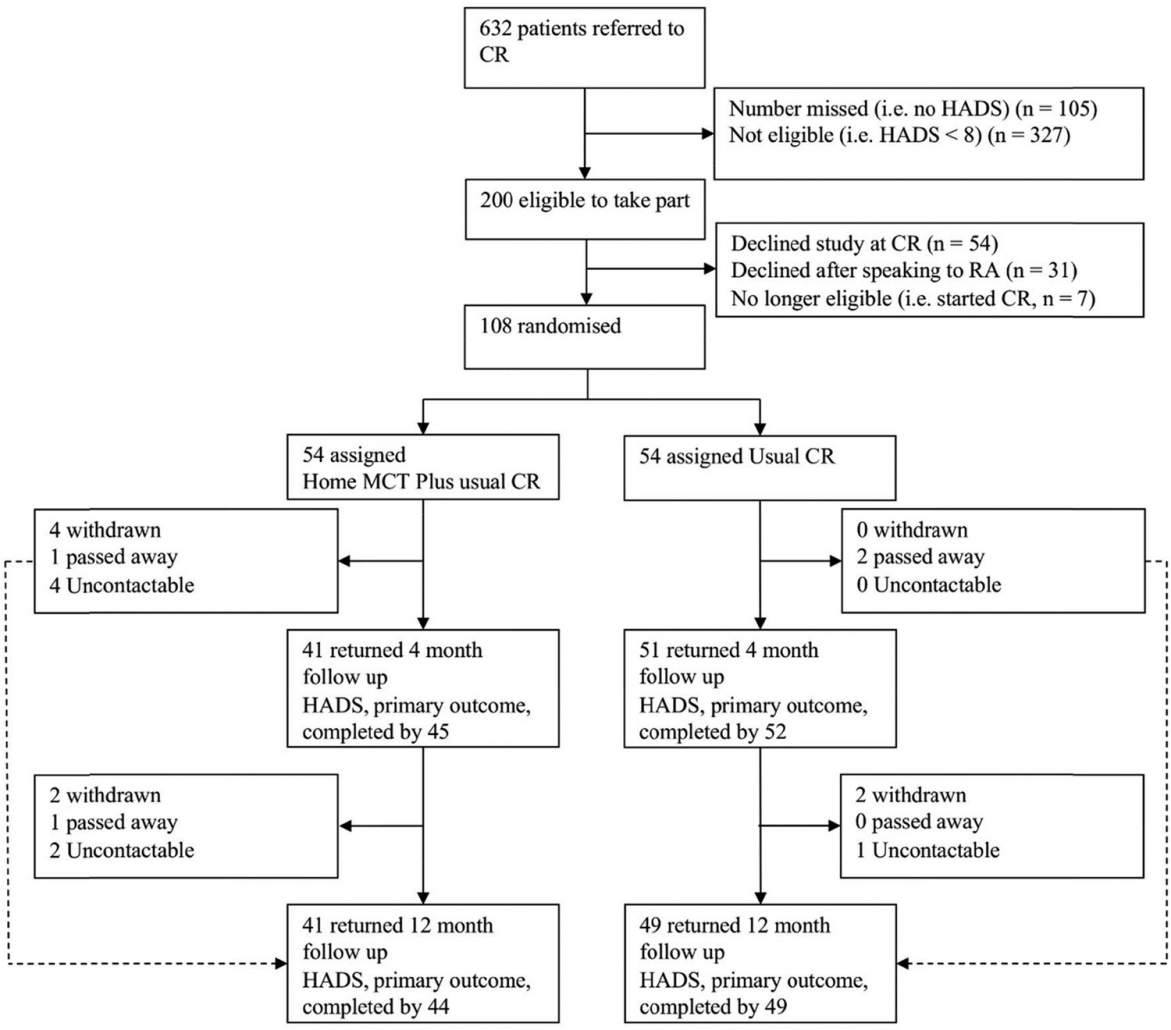
Consolidated Standards of Reporting Trials (CONSORT) Diagram of Patients in Cardiac Rehabilitation (CR) *Note*. HADS = Hospital Anxiety and Depression Scale; RA = research assistant; MCT = metacognitive therapy.

The Consort diagram of patient numbers and flow is depicted in Figure 1. One hundred eight participants (69 males, 39 females) took part in the study (i.e., were randomized). Patients had a mean age of 59.9 years (*SD* = 9.7, range = 40–84). Participants’ ethnic origin was primarily White (96.3%); though four identified as: Asian or Asian British Indian (*n* = 2), Asian or Asian British Pakistani (*n* = 1), and Black or Black British African (*n* = 1). Patients had a range of heart conditions including acute coronary syndrome (*n* = 80), revascularization (*n* = 33), heart failure (*n* = 15), angina (*n* = 2) implantation of cardioverter defibrillator (*n* = 2), and heart valve repair/replacement (*n* = 7). At initial assessment, 43 patients met criteria for both anxiety and depression (defined as a HADS score of 8 or more on both subscales), 52 patients met criteria for anxiety only, and 13 met criteria for depression only. Table 1 provides details of participant demographics and scores on measures at baseline.[Fig fig1][Table tbl1]

### Feasibility Assessment

#### Recruitment

Six hundred thirty-two patients were referred to CR services between April 1, 2017 and February 26, 2018; of which 200 (31.6%) were eligible to take part. One hundred eight patients agreed to participate and were consented and randomized to the study as summarized in the trial flow diagram in Figure 1.

#### Outcome Measures

**Figure 2 fig2:**
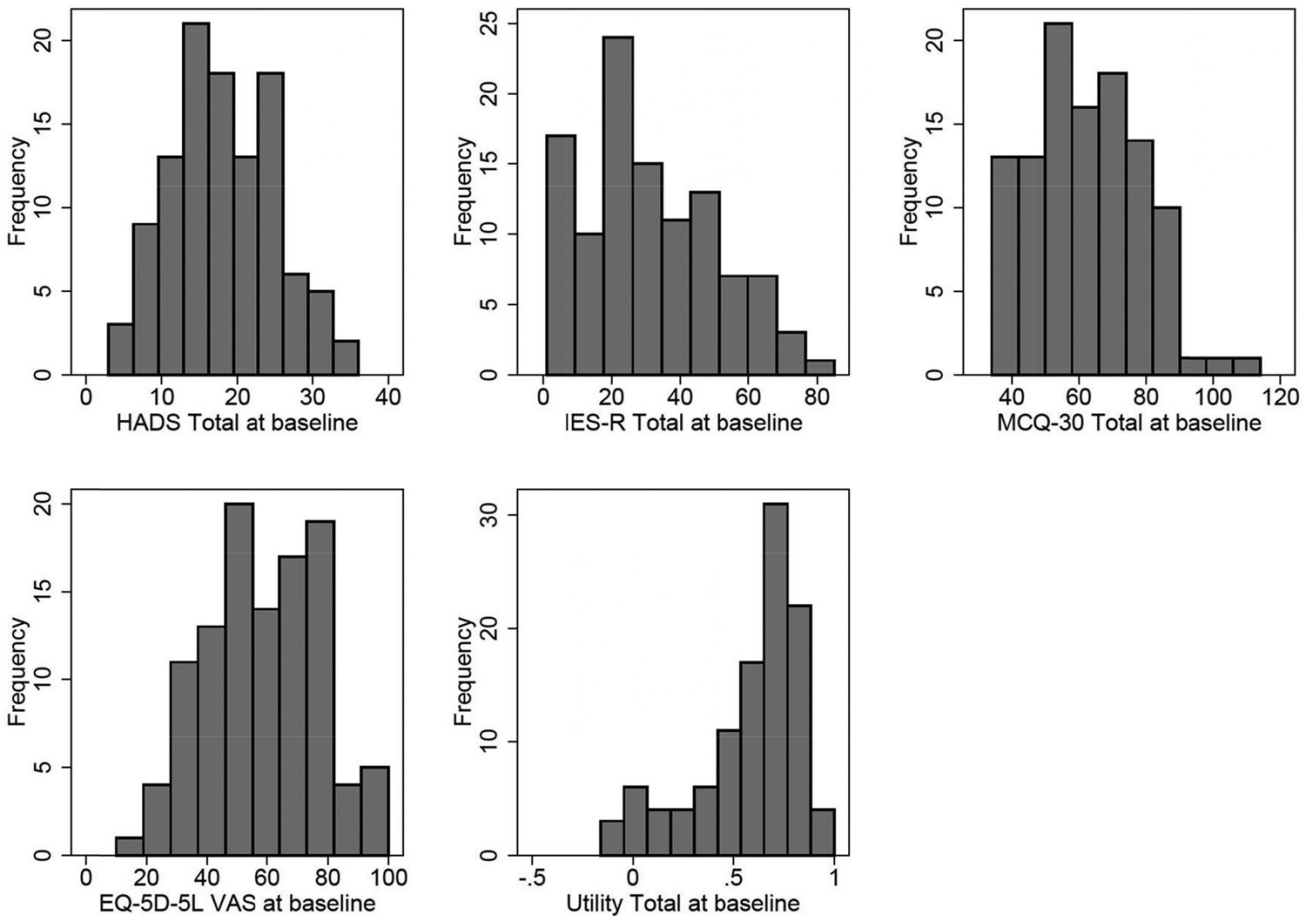
Outcome Measure Histograms *Note*. HADS = Hospital Anxiety and Depression Scale; ISE-R = Impact of Events Scale-Revised; MCQ-30 = Metacognitions Questionnaire-30.

There were no missing data on any outcome measures at baseline. Table 2 provides a summary of the descriptive statistics for each outcome measure and Figure 2 provides a histogram for each. For all scales, Cronbach’s α values indicated good reliability: HADS total = .85, HADS anxiety = .85, HADS depression = .77, MCQ-30 = .90, IES-R = .93, and CAS-1R = .82. All outcomes demonstrated a good range of observed scores, covering the majority of the possible score range, with little in the way of floor or ceiling effects. HADS scores at baseline could (and did) go below the minimum of eight that applied at the time eligibility was assessed.[Table tbl2][Fig fig2]

### Treatment Attendance and Retention

#### Control Arm (CR Alone)

Attendance at CR was high, with 89% of CR-only patients attending exercise sessions and 85% attending educational sessions. Only 11% did not attend any CR exercise sessions and 15% did not attend any educational seminars.

#### Intervention Arm (MCR + CR)

Attendance was also high for patients in the intervention arm, with 80% attending exercise sessions and 78% attending educational sessions. A slightly higher proportion of intervention group participants did not attend exercise sessions (20%), and 22% did not attend educational seminars.

#### Patient Retention

Both the control and Home-MCT arms had high retention rates at 4-month follow up. Fifty-two (96.3%) control participants provided 4-month follow-up outcome data (one returning the HADS only), with two patients unfortunately passing away before 4 months. By contrast, 45 (83.3%) Home-MCT participants returned 4-month follow up data (four the HADS only): one had passed away, four withdrew from the study and a further four did not return data. Reasons for withdrawal included no longer feeling anxious or depressed and therefore did not feel the manual was appropriate anymore, family bereavements, and two did not provide a reason for withdrawal. The overall attrition rate on the primary outcome of total HADS at 4 months was 10%.

Retention remained high at 12 months with 90.7% of control and 81.5% of intervention participants returning follow up information. The control group had two participants (3.7%) withdraw from the study and one did not return any follow-up data. The Home-MCT group had two participants (3.7%) withdraw, one was lost to death, and a further two (3.7%) did not return follow up data. Reasons for withdrawal included inability to concentrate on the questionnaires and no longer interested in taking part in the study.

#### Home-MCT Adherence

**Figure 3 fig3:**
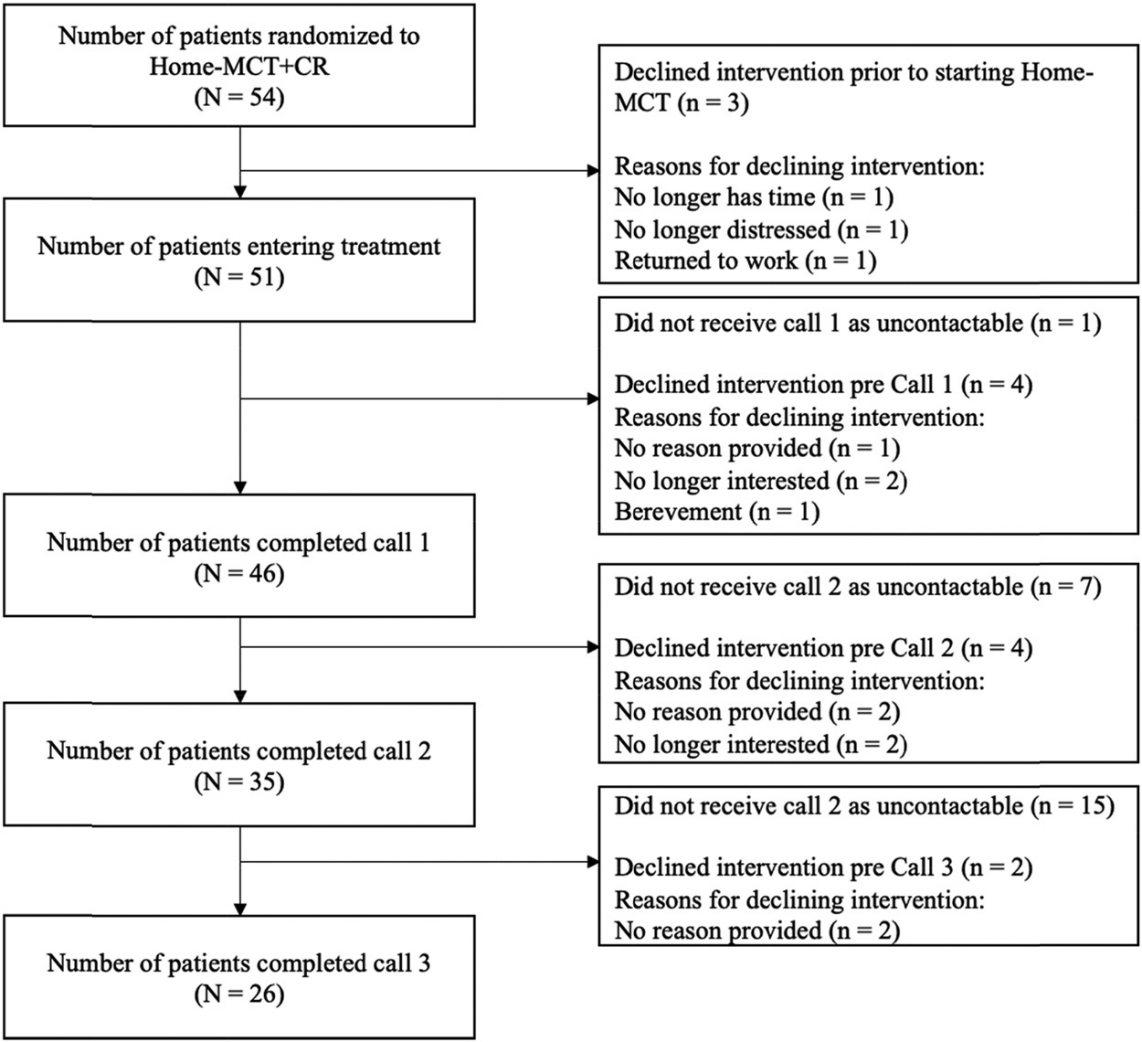
Patient Flow in Home-Metacognitive Therapy (MCT) *Note*. CR = cardiac rehabilitation.

Of the 54 patients randomized to Home-MCT, 51 patients entered treatment. Three did not begin the Home-MCT manual. Reasons for declining the intervention included: no longer felt they had time, they were no longer interested, and returning to work. For an overview of the patient flow through the intervention see Figure 3. Twenty-four patients completed at least four of the six modules - with 23 completing all six - representing 45.3% of all Home-MCT patients still alive at 4 months, or 72.7% of those who returned the credibility questionnaire. Most patients reported completing a module in 60 minutes, with individual times ranging from 40 to 105 min.[Fig fig3]

### Acceptability

Thirty-seven patients completed the credibility questionnaire, seven patients withdrew and therefore we did not expect them to return a questionnaire while the remaining 10 patients did not provide a reason for not returning the questionnaire. Overall, Home-MCT demonstrated high credibility, as noted in Table 2, 62.1% of patients rated the manual 70 or above. Many patients were initially skeptical of whether the course would help them in reducing their anxiety and depression, with only 37.8% of patients rating the usefulness as 70 or above. Despite some initial concern on the usefulness of the manual (i.e., after reading the introduction) 56.7% stated that they were likely (rating of 70 or above) to recommend the manual to a friend to help them in managing their distress.

After completing the manual patients were assessed on how user-friendly they found home-MCT, see Table 3. Home-MCT was rated highly with patients stating they found the manual easy to use and understand (median rating of 80 out of 100), that the homework was easy to follow (median rating of 85 out of 100), and that the exercise SpACE was easy to use (median rating of 90 out of 100). When asked if they found that they needed the telephone support calls, results were mixed: 40% said they did not need the support calls, while 40% stated they did.[Table tbl3]

### Home-MCT Telephone Support Calls Quality Ratings

All available telephone support recordings were quality assessed, except where the sound was inaudible. This included 29 call two (out of the 35 completed calls) and 25 call three telephone support calls (out of the 26 completed calls). Reasons for not receiving a telephone support call are detailed in Figure 3. For call two recordings, quality ratings were low for both treatment components (SpACE and worry delay), with a median of 25% (implemented poorly) on both items. Interrater reliability (Cohen’s κ) was high for both components: κ = 1.00 (*p* < .001), *n* = 29 and κ = .94 (*p* < .001), 95% CI [.49, 1.00], *n* = 29, respectively. Call three Quality ratings were similar with a median of 25% (implemented poorly) for SpACE, worry delay, and the behavior prescription elements. Kappa ratings were high for each element: κ = .80 (*p* < .001), 95% CI [.45, 1.00], *n* = 24; κ = .79 (*p* < .001), 95% CI [.54, 1.00], *n* = 25; κ = .74 (*p* < .001), 95% CI [.44,.94], *n* = 25, respectively. Only one element (uncontrollability) received a higher rating with a median of 50 (implemented moderately). Interrater reliability remained high among both raters, κ = .81 (*p* < .001), 95% CI [.60, 1.00].

### Study Eligibility Rates Before Baseline

Patients experienced an unpreventable delay between their CR assessment appointment and study baseline assessment. The average delay was 10.3 days (*SD* = 9.0; range = 0 to 55); however, study baseline assessments were always conducted before a patient’s first CR session (i.e., first exercise class). HADS scores may change over this period, with some patients no longer being within the eligibility criteria of eight points on the anxiety or depression subscale, and some may experience spontaneous clinical recovery. We examined the change in HADS scores between initial assessment and study baseline. Clinical improvement was defined as an eight-point reduction on the HADS, and clinical recovery was defined as an eight-point change and crossing the cut-off score, as calculated using the Jacobson and Truax (1991) reliable change criteria. Based on this, a small number; seven patients (6%) made a clinically significant improvement, and two patients were classified as recovered between assessment and baseline.

### Sample Size for a Full Trial

The Pathway home-MCT RCT is a superiority trial comprising of an initial feasibility trial (reported here) followed by a main trial. The main trial was designed to detect an effect size of .4 on HADS total at 4-month follow up at 90% power. Under assumptions of 20% attrition and a .5 correlation between baseline and 4-month follow up, we provisionally estimated that a total recruitment sample of 246 patients would be required for the main trial, subject to revision based on the findings of this feasibility study. In the event, this study had an overall attrition rate of 10% at 4 months and a baseline to follow-up correlation of .58. However, there was evidence for greater attrition in the home-MCT group at 17%, so for conservative reasons we choose not to revise by downsizing the sample size estimate for the main trial.

### Adverse Events

Adverse events and serious adverse events were monitored for individuals in the intervention group. No adverse events were reported. For further details on the safety reporting see the trial protocol (Wells et al., 2018).

## Discussion

Anxiety and depression are common after a cardiac event; however, current psychological interventions are limited in efficacy. In addition, most psychological interventions are offered in a face-to-face format that may be a barrier for CR patients who are unable to attend face-to-face treatment or may be returning to work. The current study aimed to evaluate the acceptability of self-help home-MCT in cardiac patients and to evaluate the feasibility of conducting a full-scale randomized trial comparing home-MCT plus usual CR to usual CR alone in this patient population.

Findings regarding the acceptability of home-MCT were somewhat mixed. Just under 50% of patients allocated to home-MCT self-reported that they completed four modules or more. However, we lacked participation data for patients who dropped out or did not return the credibility questionnaire: of those that did return it, over 70% completed the entire home-MCT course. These patients also indicated that they felt home-MCT to be credible and acceptable, with the manual easy to follow, easy to use, and easy to understand. There were no adverse events reported associated with the treatment. If we assume a 50–70% completion (of at least four modules) this compares favorably against other forms of self-help, Lundgren et al. (2016) evaluated guided web-based CBT for heart failure therapy with depression (*n* = 50). They found that 60% of patients completed four out of the seven modules, but only 24% completed all seven modules. In mental health settings, Fleming et al. (2018) conducted a systematic review of digital self-help interventions (*n* = 11) for depression and anxiety but only two studies reported completion rates (Menzies et al., 2016; Neil et al., 2009). Neil et al. (2009) noted that 2.8% of patients completed all five modules, while Menzies et al. (2016) noted that 19.5% of patients completed all seven modules.

Patients were evenly split on the necessity of telephone support calls. In addition, the delivery quality of the support calls was predominately rated as poor, with only one component (challenging uncontrollability and danger on call two rated as moderately well implemented. These results suggest that in future patients could be offered a choice of receiving the telephone support calls. Further development should be focused on improving the quality and consistency of support given by modifying the support transcripts and training in their use. This should include the introductory telephone calls, to aim to maximize patient motivation to engage with the intervention and to participate in the data collection processes.

The study results support the feasibility of delivering a trial of home-based metacognitive therapy within CR services. The addition of home-MCT to CR did not impact on delivery of CR as usual, as there was high attendance at CR exercise classes and educational sessions in both arms. In addition, retention remained high with 96% of the control patients and 83.3% of home-MCT patients completing the HADS primary outcome measure at 4-month follow-up, while 90.7% and 81.5%, respectively, completed it at 12 months. To encourage patients to complete questionnaires, we incorporated a range of strategies including sending newsletters in between follow-up time points to ensure continued contact with the study, telephoning patients to remind them that they were due to receive a follow up pack, reminder telephone calls, and incentivizing return of questionnaires. In addition, we also provided patients with a range of options for completing measures that included completing questionnaires over the phone and face-to-face with a research assistant.

The results of the present study compare favorably with previous studies of self-help interventions for psychological distress in patients with physical illnesses. Beatty and Lambert’s (2013) review of 24 self-help interventions noted that the attrition rates were variable. Attrition, defined as the percentage of participants who did not provide follow-up data, ranged between 7.6% and 56%. The overall attrition rate in our study was 10%, but was higher in the home-MCT arm at 17%, indicating that some work to understand the reasons and mitigate them may be required.

In relation to our initial research questions, we found that home MCT is feasible to deliver in CR services, with clear evidence of high levels of recruitment and retention. The intervention was found to be an acceptable treatment for anxiety and depression, as indicated by high levels of adherence to the manual, high number of modules completed, and exit questionnaire ratings. However, the reaction to telephone support was mixed and quality of calls was rated low. Initial credibility on reading the introduction of the manual was lower than expected. The preliminary statistical analysis confirmed our initial sample size estimate for a full-scale randomized controlled trial and supported our selection of measures to sufficiently discriminate between patients.

In conclusion, an intervention trial using home-based MCT appeared to be feasible and acceptable to most CR patients in the study, but further research is needed to determine if it can be rolled-out elsewhere as successfully. The results indicate that a planned randomized trial of home-MCT within CR does not impact negatively on attendance at usual CR. However, future analysis should evaluate if the supportive telephone calls are necessary. The results of this feasibility study were reported to the trial steering committee and the funder (NIHR), and a decision to extend recruitment to a full-scale RCT incorporating the feasibility study data was supported (trial registration: NCT03999359).

## Figures and Tables

**Table 1 tbl1:** Baseline Demographic Characteristics and Scores on Measures

Demographic factors	Entire sample (*N* = 108) *n* (%)
Sex	
Male	69 (63.9%)
Female	39 (36.1%)
Ethnicity	
Any White	103 (95.4%)
All other categories	5 (4.6%)
Psychological therapies for anxiety or depression	
In the past	71 (65.7%)
Never	37 (34.3%)
Age [*M*(*SD*)]	59.9 (9.7)
Employment	
Economically active	44 (40.7%)
Unemployed	11 (10.2%)
Retired	40 (37.0%)
All other	13 (13.1%)
Educational qualification	
None	23 (21.3%)
School/vocational	57 (52.8%)
Diploma/degree	28 (25.9%)
Civil status	
In a relationship	65 (60.2%)
Separated	3 (2.8%)
Divorced	10 (9.3%)
Widowed	11 (10.2%)
Single	18 (16.7%)
Do not wish to disclose	1 (0.9%)
Smoking status	
Never smoked	25 (23.2%)
Ex-smoker	71 (65.7%)
Current smoker	12 (11.1%)
Alcohol units per month	
None	30 (27.8%)
1 to 19	55 (50.9%)
20 to 49	10 (9.3%)
50 or more	13 (12.0%)
Age at first cardiovascular event	
Under 45 years	17 (15.7%)
45 to 54 years	37 (34.3%)
55 years and older	54 (50.0%)
Number of previous cardiac events	
None	1 (0.9%)
1	79 (73.1%)
2 or more	28 (25.9%)
BMI	
Underweight/normal	15 (13.9%)
Overweight	31 (28.7%)
Obese	62 (57.4%)
Number of comorbidities [*M*(*SD*)]	3 (1.7)
Symptom/process measures	Mean (*SD*)
HADS total	18.3 (7.0)
HADS anxiety	10.4 (4.4)
HADS depression	7.9 (3.9)
Impact of Event Scale-Revised (total)	31.6 (19.3)
Metacognitions Questionnaire 30 (total)	61.6 (16.0)
EQ-5D-5L (VAS)	58.0 (19.3)
EQ-5D-5L (utility score)	0.58 (0.26)
*Note*. BMI = body mass index; HADS = Hospital Anxiety and Depression Scale.	

**Table 2 tbl2:** Descriptive Statistics on Outcome Measures at Baseline

Outcome measure	Sample size	% Missing	Median (interquartile range)	Minimum and maximum observed scores	Minimum possible score (% scoring minimum)	Maximum possible score (% scoring maximum)
Hospital Anxiety and Depression Scale (total score)	108	0	18 (13 to 23)	3, 36	0 (0%)	42 (0%)
Impact of Event Scale-Revised (total)	108	0	28 (18 to 44.5)	1, 85	0 (0%)	88 (0%)
Metacognitions Questionnaire 30 (total)	108	0	59 (50 to 73.5)	34, 114	30 (0%)	120 (0%)
EQ-5D-5L (VAS)	108	0	60 (45 to 75)	10, 100	0 (0%)	100 (1%)
EQ-5D-5L utility score	108	0	0.66 (0.48 to 0.76)	−0.16, 1	−0.594 (0%)	1 (3%)

**Table 3 tbl3:** Intervention Acceptability and Adherence Outcomes

Questionnaire	Question	*Mdn* (IQR)/yes (%)	*N*
Credibility	How logical does the Home-MCT manual offered to you seem?	70 (60 to 90)	37
	How successful do you think the Home-MCT manual will be in reducing your distress?	60 (50 to 80)	37
	How confident would you be in recommending the Home-MCT manual to a friend	70 (50 to 90)	37
Acceptability	How many modules of the Home-MCT have you completed?	6 (1 to 6)	37
	I found the Home-MCT easy to use?	80 (50 to 90)	32
	I found the Home-MCT easy to understand	80 (50 to 90)	31
	I found the homework easy to follow	85 (50 to 90)	32
	I found the SpACE easy to use	90 (40 to 100)	31
	I found that I needed the supportive telephone calls	65 (10 to 90)	30
End of intervention	Module 1 completed	28 (77.8%)	37
	Module 2 completed	27 (79.4%)	37
	Module 3 completed	27 (79.4%)	37
	Module 4 completed	24 (72.7%)	37
	Module 5 completed	23 (71.9%)	37
	Module 6 completed	23 (71.9%)	3737
	No modules completed	9 (24.3%)	
	Module 1 time in minutes	60 (50 to 105)	31
	Module 2 time in minutes	60 (50 to 85)	29
	Module 3 time in minutes	60 (30 to 75)	29
	Module 4 time in minutes	60 (45 to 85)	27
	Module 5 time in minutes	60 (45 to 85)	26
	Module 6 time in minutes	60 (40 to 90)	26
*Note*. MCT = metacognitive therapy.
